# Factors influencing pregnancy planning of multi-ethnic Asian women with diabetes: A qualitative study

**DOI:** 10.1371/journal.pone.0242690

**Published:** 2020-12-03

**Authors:** Irmi Z. I., Ng C. J., Lee P. Y., Hussein N.

**Affiliations:** 1 Faculty of Medicine, Department of Primary Care Medicine, University Malaya, Kuala Lumpur, Malaysia; 2 Faculty of Medicine and Health Sciences, Department of Family Medicine, Universiti Putra Malaysia, Serdang, Selangor, Malaysia; Makerere University, School of Public Health, UGANDA

## Abstract

**Introduction:**

Pregnancy planning varies among women with diabetes. Observing that the literature examining the factors affecting diabetic women’s pregnancy intentions in multi-ethnic Asian populations is limited, we sought to explore these factors to give a better perspective on these women’s pregnancy planning.

**Methods:**

This qualitative study used individual in-depth interviews to capture the views and experiences of non-pregnant diabetic women of reproductive age in four public health clinics in a southwestern state of peninsular Malaysia from May 2016 to February 2017. The participants were purposively sampled according to ethnicity and were interviewed using a semi-structured topic guide. Interviews were audio-recorded, and transcripts were analysed using thematic analysis.

**Results:**

From the 33 interviews that were analysed, four important factors influencing participants’ decisions regarding pregnancy planning were identified. Participants’ perception of poor pregnancy outcomes due to advanced age and medical condition was found to have an impact. However, despite these fears and negative relationships with doctors, personal, family and cultural influences supported by religious ‘up to God’ beliefs took centre stage in the pregnancy intention of some participants. Participants demonstrated a variety of understandings of pregnancy planning. They outlined some activities for pregnancy preparation, although many also reported limited engagement with pre-pregnancy care.

**Conclusions:**

This study emphasised the known dilemma experienced by diabetic women considering their desire for an ideal family structure against their perceived pregnancy risks, heterogeneous religious beliefs and the impact of cultural demands on pregnancy intention. This study urges healthcare providers to increase their engagement with the women in pregnancy planning in a more personalised approach.

## Introduction

Diabetes in women of reproductive age has become a major public health concern in Asia because of the higher prevalence of diabetes among adult women than among their male counterparts [1–4]. The world is observing increasing numbers of child deliveries by mothers with pre-existing diabetes [4,5] and appreciating the financial liability that diabetic pregnancies represent for the healthcare system [6,7]. Therefore, efforts to optimise diabetes control as part of pre-pregnancy care (PPC) as the key strategy for better pregnancy outcomes are becoming ever more relevant in the current healthcare setting [8,10]. However, despite the many international and local guidelines on PPC for diabetes [8–12], diabetic women still embark on unplanned pregnancies [11–15]. Globally, the uptake of PPC by diabetic women ranges between 27.5%–58% in developed countries such as Australia [11], the United Kingdom [12], the United States of America [13] and Denmark [14]. In Malaysia, a study conducted in a university hospital showed that more than 50% of post-partum women who had not planned their pregnancy had pre-existing diabetes [15].

A planned pregnancy is a pregnancy which is desired, well-timed, agreed to by both partners and preceded by PPC [16]. Extensive lifestyle preparations and consultations with a doctor are some steps of a planned pregnancy that have been demonstrated as protective factors against bad pregnancy outcomes for women with pre-existing diabetes [9,17]. Even though the concept of planning for a pregnancy is familiar to healthcare providers, women may understand it differently [16]. They hold particular beliefs around fertility and, despite the risks that they incur because of diabetes, pregnancy may be socially significant to them and their family [18]. Therefore, it is imperative to clarify the women’s intention and their needs regarding pregnancy in order to best prepare them for their pregnancy. Many of the studies on the factors influencing diabetic women’s intention to get pregnant are carried out in Western countries, and data from Asia is limited. In view of the differences in social and cultural backgrounds, the factors influencing diabetic women’s pregnancy intentions in Asian countries like Malaysia may vary. Similarly, their preparations for pregnancy may differ. Consequently, we sought to explore the factors influencing pregnancy planning and understanding of PPC among diabetic women in Malaysia to give a richer perspective on how PPC can best be delivered to them.

## Materials and methods

### Study design and participants

The present research consists in an interpretive and descriptive qualitative study, which captured the pregnancy planning experiences of non-pregnant women of reproductive age who were on follow-up at diabetic clinics in four government primary care clinics in a southwestern state of peninsular Malaysia. This state was chosen in view of its history of diabetic prevalence since 1996, despite having a population of only 1.12 million [19]. Participants were purposively sampled according to Malaysia’s three major ethnic groups, age (under 45 years old), pregnancy intention or lack of as well as locality. Two urban and two rural clinics with existing pre-pregnancy registry services were chosen from the districts with the highest and lowest population density [19].

Potential participants identified from the pre-pregnancy registry or the list of scheduled diabetic appointments were approached and invited to take part in an interview on the day of their appointment. Menopausal women and those who did not have an active life partner, were not cognitively stable or were unable to converse in Bahasa Malaysian or English were excluded. This study was approved by the National Medical Research Ethics Committee of the Ministry of Health of Malaysia in April 2016 (NMRR ID: 16-385-29240).

### Data collection

Data collection, including data analysis, took place between May 2016 and February 2017. Individual in-depth interviews (IDI) were employed because relating experiences regarding pregnancy can be a sensitive issue for some women and to protect the participants’ privacy. To ensure consistency between interviews, a single researcher (II) conducted all the IDIs. II is a female primary care physician who had never worked at the study clinics before. All researchers (II, NCJ, LPY and NH) are primary care physicians and medical lecturers and have experience in qualitative research and in interviewing patients in clinical research settings. The team’s clinical experience in dealing with diabetic women at public clinics enabled them to understand the flow of the organisation and make sense of the participants’ reflections. II only sought clarification using the participants’ own words so as not to sound like a clinician and trigger any bias in the participants’ responses.

The participants were briefed about the study and the need to audio-record the interview. They were given assurances regarding data confidentiality and the protection of their privacy through the use of pseudonyms during the interviews, in the transcripts and in any future publications. They were also assured that the interview will have no consequence for their future diabetic management. Once written consent was secured from each participant, interviews were carried out either on the same day in a more private area of the clinic, or rescheduled to the convenience of the participant.

Interviews were guided by a semi-structured protocol combining the theory of reasoned action and the theory of planned behaviour [20–22]. The topic guide (S1 Appendix) consisted of three important aspects of pregnancy planning among women with diabetes:

1. Influences on pregnancy intention;
2. Understanding of PPC;
3. Experiences in their preparation to conceive.

In addition, the IDIs also took into account other background factors, such as past history of planning for pregnancy, socio-demographic factors and use of family planning. Family planning users are women who were using modern and effective contraception at the time of the study. A semi-structured guide loosely outlined the topics and allowed for flexibility.

### Data analysis

The interviews, which lasted between 40 and 60 minutes, were audio-recorded and transcribed verbatim. The data was organised and analysed using NVivo 10 software. The research team then carried out thematic analysis [23]. The authors familiarised themselves with the data by repeatedly listening to the audio-recordings and reading the written transcripts. The team members (II, NCJ, LPY and NH) analysed the data inductively and coded the first three transcripts independently. Codes, which were short texts of concepts that emerged from the transcripts, were collated into categories and subsequently into themes based on their similar meaning and concept. Codes with similar concepts were later categorized into themes [23]. The codes were constantly compared across different interviews and all the team members reviewed, discussed the themes back and forth during meetings before finally agreeing on the final themes. Observations on participants’ expression and interviewer’s feelings gathered from the field notes were given appropriate consideration to verify the participants’ responses and add credibility to the results. These themes serve as the main framework for subsequent coding during which new themes if any, are added on to it. Disagreements on certain codes were solved by re-examining the meaning of the codes and referring to the objectives. This rigorous analysis ensured that the meanings of the codes and the objectives of the study were preserved.

Efforts were made to contact as many as 23 participants who were deemed to represent the women’s various intentions regarding pregnancy for a member’s check. However, only 12 attended the session. Some did not answer to the calls (n = 8), whereas others did not turn up for their appointments (n = 3). The documents containing the summary of transcript findings, coding and excerpts were given to the participants during the member check. The participants were given time to read through the documents, confirm the findings and inform the team of any need for modification or addition [24,25].

A point of saturation was reached when no new theme was unearthed. The data was saturated at the 27th participant for two themes; health concerns and the relationship between healthcare practitioners and women with diabetes. By participant 33, no new information appeared regarding the ‘up to God’ belief and desire for an ideal family. Thus, no further coding could be done to elicit any new theme.

## Results

The team invited 40 women to participate; 33 were effectively interviewed. Five were excluded because they were menopausal, and another two refused to participate. The median (IQR) age of the participants was 37.0 (7.5) years old, and the majority were between 30 and 40 years old. Except for two women aged 43 and 40 years old, all other nulliparous participants or participants with no living child were younger women. They worked as housewives (n = 9), in business (n = 9), as general workers (n = 6), clerks or in office-related jobs (n = 6), in the medical sector (n = 2) and as teachers (n = 1). Out of the 11 women with pregnancy intention, nine had poor blood sugar control. There were almost similar number of urban and rural participants. Participants did not highlight locality as a theme to influence their pregnancy planning. S1 Table presents the background data on the participants.

### Pregnancy intention

Out of 33 women interviewed, 11 participants had intended to pursue a pregnancy within 6 months. Ten were nulliparous or had no living child. All women of advanced age (>35 years old) who had no living child showed intention for pregnancy (n = 6). Another four participants displayed ambivalence towards pregnancy, while the remaining 18 participants were quite firm about having no current or long-term pregnancy intention. During the member’s checks, the participants stood by their previously voiced pregnancy intention, except for one person, making her a unique case in this study. Indeed, participant S5 changed her view on her pregnancy planning. She initially had very strong pregnancy intention despite her poorly controlled diabetes and having aborted once. After being hospitalised for an ectopic pregnancy, she stated how important it was for her not to get pregnant and made vast efforts to use contraception while stabilising her diabetes.

### Factors influencing pregnancy intention in diabetic women

We identified four important factors that influenced the participants’ decision in favour of or against pregnancy:

1. Participants’ perception of poor pregnancy outcome due to advanced age and complications related to their medical condition;
2. Participants’ desire for an ideal family structure;
3. ‘Up to God’ belief;
4. Role of doctors.

#### Perception that advanced age entails higher pregnancy risks

The participants remarked that they are already at an advanced age or approaching one. They had differing opinions on what constitutes advanced age, but most proposed 35 years old and above. Regardless of their parity, the perception that they were at an advanced age prompted some women to avoid pregnancy. They felt that their physical capacity to go undergo another pregnancy at their age was reduced and that a pregnancy would complicate their life.

*“I’m still 38*… *not 40 yet*, *right*? *Ah*… *they advised that because we are getting old*, *right*. *So*, *if we were to be pregnant*, *I feel more troubled*. *I’m feeling very tired nowadays… tired*. *If I don’t take medicine*, *I felt giddy*… *Always feeling that way*… *So*, *no [pregnancy]*! [Participant K2, 38 years old Indian, Para 2, no pregnancy intention, has poor sugar control and practise withdrawal method]

“*If we become pregnant at the age of 35*, *we know that anything can happen*. *Baby getting down syndrome and abnormal*. *So I do not gave high hopes for pregnancy anymore*.” [Participant S, 32 year-old Malay, P0+1, ambivalent towards pregnancy, good sugar control, not on contraception]

Another factor that discouraged them was their fear of age-related foetal abnormalities complicating the pregnancy. The participants were aware of age-related foetal abnormalities such as Down Syndrome, and they expressed concern that they would not be emotionally capable to handle raising abnormal children. Indeed, the thought of caring for their young at an advanced age, even if they are born normal, was also a concern to them.

*“Im now 34 years old*. *I don’t think I want to try (conceive) again after this 2 years (after 36 years old)*. *It is actually quite difficult to go through*. *It is not about me…Let say if I have a baby later (at older age)*, *abnormal*. *I don’t know I can handle it or not*. [Participant S6, 34year old Chinese, Para 1+2, has intention for pregnancy in 6 months, poor sugar control and on condom]

#### Perception of their current medical condition and related risks for pregnancy

Many of the participants were aware of the pregnancy risk associated with diabetes, which influenced their intention not to conceive. They perceived diabetes as an unhealthy state, a disease that would worsen their health if they became pregnant, and more so if their diabetes was poorly stabilised. Even though they were concerned that the disease may complicate their lives during pregnancy and during and after delivery, the utmost concern relayed by many participants related to the ill effect of diabetes on the foetus. They worried that their babies would suffer from complications, such as foetal abnormalities, prematurity or stillbirth. Majority with no pregnancy intention (n = 15/19) has poor sugar control.

*‘I feel that if I can’t control my sugar and if I conceive when it is high*, *maybe my baby would be abnormal…aaa I know that the effect on baby is very bad*. *And I have to be admitted frequently*..*so at the moment I’m not planning to get pregnant’* [Participant S4, 37 Malay, Para 2, no intention for pregnancy, has poor sugar control but not practising any contraception]

A few participants believed that diabetic pregnancy predisposes the baby to inherit the disease or contract it early in life. The belief came from their life experience and observations.

*“I think it is because my mother in law has diabetes and all four children have diabetes*. *That is what I think la*. *My husband also has diabetes*. *Very troublesome*. *So Im scared*..*if I really deliver and my child got it*. *He would have a lot of trouble too*.*”* [Participant J7, 42 Chinese clerk, Para 2, no pregnancy intention, good sugar control, practising abstinence]

Experience in stabilising diabetes before and during a previous pregnancy, whether first-hand or observed, may have had a profound emotional effect on some participants’ decision not to get pregnant. For example, participant R6, whose husband requested another baby, shared the difficulties and the emotional burden she felt having to control over her blood sugar level. Another participant, participant S5 initially had a very strong pregnancy intention despite acknowledging the pregnancy risks. However, the daunting experience of her recent hospitalisation for an ectopic pregnancy shifted her attitude from gambling on the risk to a more cautious approach. She even took steps to improve her practice of contraception, with the aim of delaying pregnancy while stabilising her diabetes.

*“My husband wants another kid*. *He knows I have diabetes*. *I don’t feel good knowing that he wants another kid*. *But Im also on insulin*, *every night I inject insulin on my tummy*. *That is why I don’t think of having more babies*. *My sugar is also high*..*Enough*! *Im not thinking about it*.*”* [Participant R6, 33year old Indian, has one child, no intention for pregnancy, poor sugar control but not practising contraception]

*“I feel that having a child completes me*. *I still want a child*. *Without one I don’t feel excited*. *But when I got ectopic pregnancy*..*Im scared*. *So I want to defer*. *Maybe I wasn’t aware before this…The incidents in the ward changed my perspective”*[Participant S5, 27 year old, Malay, Para 0 with 2 abortions, ambivalent towards pregnancy, has poor sugar control and avoid sex during fertile period]

#### Desire for an ideal family structure

The desire to expand the family, have one or more children contributed heavily to the pregnancy intention of many participants. Out of eleven who had the intention to pursue a pregnancy, ten did not have a living child. Participants had different reasons to expand their family; most were linked to perceived biological need to be a mother, but the influence of family members, husbands, mothers-in-law and even existing children had a more profound effect. One participant (participant J8), who feared that without a child of her own, her blissful marriage was in danger, underwent seven abortions before finally delivering one girl.

*“Haa…my mother in law doesn’t have that many children*. *My husband has only 2 siblings*. *So she wants grandchildren*. *Im sad that I cannot give her one*. *So when I have diabetes*, *I think surely my husband will marry another woman*. *And he will leave me*. *So…that is what I felt*. *What am I going to do*? *He is rich*, *I don’t have anything*. *I only have him”* [Participant J8, 40 year old,Malay, Para 1 and 7 abortions, has no pregnancy intention, poor sugar control and avoid sex during fertile period]

#### ‘Up to God’ belief

The participants held an ‘up to God’ belief in varied ways and gave it varied meanings, with different outcomes on pregnancy intention. During the first interview, participant S5 interpreted becoming pregnant as a sign from God that it was fine for her to embark on a pregnancy despite her current unfavourable circumstances. To her, pregnancy was God’s gift and she was not in a position to reject it. She even prayed and believed that God would stabilise her diabetes during a pregnancy.

*Now*, *is a good time (to conceive) but no luck yet*. *Maybe if Allah give me the gift*, *I cant possibily reject right*, *So if he has granted me pregnancy*, *co need to control la (diabetes)*. *If that is what Allah wish*, *then I will get pregnant*. *Haa…so I pray that who knows when Im pregnant*, *my diabetes is stabilised*. *We never know right*? *Hope la*.*”*[Participant S5, 27 year old, Para 0 with 2 abortions, ambivalent towards pregnancy, has poor sugar control and avoid sex during fertile period]

Participant J3 had a different interpretation of the ‘up to God’ belief. She also believed that pregnancy was at the hands of the Creator; nevertheless, she had no intention to conceive at that moment. As a result, she felt guilty because she was blessed with the ability to conceive but did not want to do so. She justified her viewpoint by quoting a religious standpoint according to which God admonished not to proceed with any action that would do harm to mankind and explained that conceiving while her diabetes is uncontrolled would endanger her own health.

*‘Allah is a better planner*. *Ive taken contraception before but I still got my no 3 child…God has given me a gift*, *so I accept*. *It is fated*. *But sometimes I feel if I plan not to conceive is as if I reject God’s rules because I am given the ability to conceive*. *Then again I have good reason to plan*. *Quran says do no harm to yourself right*, *so that is why I plan la*.*’* [Participant J3, 31 year-old Malay housewife, no pregnancy intention, poor sugar control and on depot provera]

#### The role of doctors in decision-making

Four participants relayed their experience of consulting their doctors regarding their intention to conceive. Overall, the experiences that they shared were not helpful and made them shy away from further discussing planning for a pregnancy with their doctors. Participants felt discouraged from disclosing their pregnancy plans to their doctors. Participant J2 perceived the doctors’ negative reactions as harsh. She felt stressed and dejected because of the ill treatment that she received with regards to her pregnancy intention. Eventually, she withdrew from future consultations and communication with the doctor. Fortunately, she still hopes that a new will come by and rekindle her pregnancy intention.

*‘Sometimes these doctors are…very harsh*. *I sometimes don’t feel like asking anything*. *The language they use is quite extreme*. *So I don’t feel like asking advice*. *There is this one specialist who said” Why get pregnant if your baby ends up dead*? *It would be better if you don’t have any*.*” Ahh*! *So I just kept quiet*. *No need ask anything*. *Until a new doctor comes then we will see how*..*’* [Participant J2, 35 years old Malay general worker, Para 0+2, uncontrolled sugar, has pregnancy intention, and not on contraception]

Similarly, participant R8 shared how crestfallen she felt when the consultation she had long waited for resulted in rough rejection from the doctor. She described feeling mortified when the doctor flung her file in front of her and bluntly concluded that nothing else could be done for her and her husband. This dissuaded her from consulting any doctor in the future.

*‘He went through my husband’s file and flung it in front of me*, *said that you cannot get pregnant anymore so go get a foster child*. *I felt very upset and cried*. *How could this doctor*, *a specialist*, *talk to me like this*? *He threw the file at me*. *I’m not seeing any doctor anymore*.*’* [Participant R8, Indian, 39 years old, Para 0, has pregnancy intention, poor sugar control and no contraception]

Another participant, participant R3, shared her experience of a less domineering consultation, which nevertheless made her shy away from seeking advice on planning for a pregnancy. The doctor focused on her uncontrolled diabetes and discouraged her from conceiving before her blood-sugar level had been stabilised. However, she did not share her main concern with the doctor, which was to find out her current fertility status rather than to request treatment to conceive. Knowing that her diabetes was poorly controlled and that the doctor would not support her pregnancy intention, she coped with her own frustration by keeping silent.

*Since I’m not stabilised yet*, *he didn’t seem to encourage me to get pregnant*. *Don’t encourage*. *But I want… at least to know whether my hormone is ok*… *Can I go to the hospital and ask for fertility check*? *Didn’t ask the doctor*. *Seems like he will not let me get pregnant*, *ask me to stabilize first*. [Participant R3, 32 years old, Indian, Para 1, no pregnancy intention and on oral contraception]

### Understanding of pre-pregnancy care

Participants showed different levels of understanding regarding PPC; some were more aware than others. Some participants had non-existent or limited knowledge on how to prepare for pregnancy or PPC. Although they did not have a great understanding of PPC, some of the participants felt that it was necessary to plan for a pregnancy.

*“emm…when you ask me about PCC…because it is a new thing this PCC*, *so I don’t know*..*maybe there is none*..*err*..*how do I put this*? *No planning…don’t know about pre pregnancy care…”* [Participant S1, 32 years old, Malay, Para 0+1 who is ambivalent towards pregnancy and not on contraception]

*“I don’t really know what PPC is but I think it is important but we need to see the specialist first*.*”* [Participant S7, 26, Malay, Para 0, has pregnancy intention and not on contraception]

On the other hand, participants with more knowledge highlighted the need to prepare and plan the pregnancy as well as follow a PPC plan for the sake of the child’s health.

*‘We need to plan first if we want to get pregnant*. *If conceive*, *we need to take care of the pregnancy well*, *so we need to decide whether we want a pregnancy or not*.*’* [Participant KP2, 38 year old, Indian, Para 2, no pregnancy intention, poor sugar control and on condom]

*‘Before [getting] pregnant*, *[there] needs… preparation like food… mental too*. *One needs to know*, *once pregnant*, *she has to use insulin*, *know how to check the sugar level three times*. *All this will cause stress*, *so we need to have patience*. *All for the sake of my child*.*’* [participant R4, 33, Indian, Para 2, no pregnancy intention and on oral contraception]

Additionally, some participants felt that it was not necessary to consult healthcare providers or require any intervention from doctors when they had no intention to conceive. To them, contraception seemed to be a natural option to avoid pregnancy, aside from abstinence.

*‘If you don’t want to conceive… no need [to] see [a] doctor*, *just take the pill*.*’* [participant R4, 33, Indian, Para 2, no pregnancy intention and on oral contraception]

*‘No need [to] ask la… Because the person doesn’t want any child*, *even the spouse also doesn’t want*, *why need to ask further*? *Just don’t do sex la’*[Participant R2, 37 year old, Chinese, para 1, no pregnancy intention, avoid fertile period]

### Women’s experiences on their preparation to conceive

Women with diabetes engaged in a wide range of activities in their preparation to conceive. The four themes that emerged include: 1) having limited engagement with PPC; 2) disclosing their intention to their healthcare providers; 3) adapting their activities to increase their chances of conception; and 4) stabilising medical risks.

#### Limited engagement with PPC

The women revealed that they embarked on past pregnancies without any planning or preparation. Some had never received any counselling to prepare for their pregnancy nor an invitation to go through PPC. Nevertheless, a number of participants received some form of counselling on PPC; they described those as non-personalised to their reproductive needs.

*‘My sister is also like me*, *we don’t plan them*, *just suddenly we are pregnant*.*’* [Participant KP1, 36 year-old Malay, Para 2+2, no pregnancy intention but not on contraception]

*‘Frankly*, *if I attend the DM clinic*, *I have to wait a long time for my turn*, *then he will only talk for a while*, *look at the results*. *Then he will be busy with other things and ask us to take the medicine*. *He doesn’t really give attention to pregnancy; only the diabetes and blood pressure*.’ [participant KP6, 27-year old Malay, Para 0, poor sugar control, has pregnancy intention and not on contraception]

‘*He just said control for pregnancy*. *None in detail*..*like no focus on the pregnancy*…*just like reduce weight*, *control sugar but none like what you should do to get pregnant’* [participant S1, 32 year old Malay, Para 0+1, no pregnancy intention and not on contraception]

#### Disclosing pregnancy intention to healthcare providers

Participants realised that informing their healthcare providers of their pregnancy intention was an opportunity to receive proper diabetes treatment and acknowledged the need for effective compliance. The disclosure of pregnancy intentions also leads to planning for conception.

*Oh*, *I’ve been taking medication for diabetes before pregnancy*, *so when I want to get pregnant*, *I told the doctor of my plan*. *So*, *the doctors change me to insulin […] so I use the insulin and follow the food advice*.’ [participant R4, 33, Indian, Para 2, no pregnancy intention and on oral contraception]

#### Increasing chances for conception

The participants identified strategies that they adopted to increase their chances to conceive. First, they recommended discontinuing contraception. Second, they sought help from healthcare providers for their fertility problems and found alternative ways to enhance their fertility (such as through traditional medicine) and highlighted activities meant to facilitate conception. The participants emphasised the importance of establishing fertility in their preconception activity and underlined their awareness of their fertile window.

*‘If we plan to conceive… [we] need to increase sexual activities and… stop [using a] condom and all*.*’* [Participant KP1, 36 year-old, Malay, para 2+2, no pregnancy intention and not on contraception]

*‘I will try to stabilise it [blood sugar] first*, *then… next step*, *check the necessary*. *We can then know what are the [fertility] problems… maybe my husband*, *we don’t know right*. *So*, *do both [fertility and blood sugar control] together at the same time*.*’* [Participant KP6, 27 year-old, Malay, Para 0, has pregnancy intention and not on contraception]

‘..*in order to conceive*, *I took many things*..*variety of medicine and jamu*.’ [Participant KP3, 40 year-old Malay, Para 0, has pregnancy intention and not on contraception]

#### Reducing medical risks for a safer pregnancy

Participants pointed out a few activities that they engage in to reduce the pregnancy risks associated with diabetes, hypertension and obesity.

#### a) Pre-pregnancy health check

Participants highlighted the need for pre-pregnancy health checks, to ensure that they embark on a pregnancy in a relatively uncomplicated and controlled environment. One participant showed motivation to be up to date with current information on her illness; this is possibly due to her long-standing diabetes, which she developed at a young age. This demonstrates the relevance of knowledge in adhering to positive pre-conception health-seeking behaviours.

*‘Because normally before three months*, *I will ask [the] doctor*: *‘can I have a check*?*’ So I will check my kidney result and also the HbA1c to ERR*, *I will know it before I start [preparing for pregnancy]*. *I will be sure it’s in a good condition*. *I [come] and talk with [the] doctor*.*’* [Participant S6, 34 year old Chinese, Para 1+2, has pregnancy intention in the next 6 months and currently on condom]

#### b) Stabilising medical risks

Many of the participants were aware of how medical risks affect pregnancy outcomes; thus, adherence to treatments and lifestyle changes were part of their priorities. They took many initiatives during their past pregnancies and in their current life to implement the recommended lifestyle changes. In addition to modern medicine, women with diabetes also mentioned the use of traditional complementary medicine as one of their efforts to control medical risks.

‘*The nurses said that if I want to conceive*, *my weight has to reduce*. *So I reduced my rice intake*, *and ha*! *my weight dropped*. *And I go for aerobic often*, *that is why my weight can reduce*.’ [Participant J2, 35 year-old Malay, Para 0+2, has pregnancy intention, poorly sugar control and not on contraception]

*‘Need to take care of diet*, *take supplement to reduce sugar level*. *I took the green drink called kaylin… for diabetes*, *for our body to feel more fresh*, *not easily tired and for our internal care*.’ [Participant KP9, 32 year-old Malay, Para 3, no pregnancy intention, poor sugar control and on depot provera]

#### c) Taking folate supplements and safe drugs for pregnancy

Although many participants mentioned taking folate supplements before conception, none spoke about its indications for the prevention of malformations. Instead, they referred to folate as a vitamin or attributed a fertility-enhancing function to it, describing it only as an important element when planning for pregnancy. Participants who were on anti-hypertensives were also reassured by the doctors about the safety of the drugs prescribed to them.

*‘When I plan to have pregnancy*, *the doctor [will] give me the folic acid three months before*. *Vitamins*. *That’s why I know*.’ [Participant S6, 34 year-old Chinese, Para 1+2, has pregnancy intention in 6 months and on condom]

*‘I’m on folic acid*. *I take it because I want to get pregnant*. *It helps to enhance my fertility’* [Participant S5, 26 Malay, Para 1+2, ambivalence towards pregnancy and avoid sex during fertile period].

*This yellow medicine for high blood pressure… they said even if I’m pregnant*, *I could still use this medicine… it can contro [blood pressure]l*. *There is no effect to my baby*. [Participant J6, 37 Malay, Para1, no pregnancy intention and on condom]

## Discussion

This study revealed that although women with diabetes voiced fears about the pregnancy risks associated with their condition, some of them put more emphasis on their desire for an ideal family structure, which was supported by religious and cultural beliefs. This study also showed that women with diabetes had limited knowledge about pregnancy planning and PPC, which may explain their limited engagement with PPC. Nonetheless, some participants reported positive pre-pregnancy behaviours intended to protect their health.

This present study is similar to many Western studies that portrayed the physical and emotional turmoil that women with diabetes face when dealing with pregnancy. The women had many concerns about their ability to care for a child while they are already burdened with caring for themselves [26]. As a result, the study showed that they often had more limited pregnancy intentions [26]. They felt that pregnancy may cause a disruption in their self-care routine [27]. Similarly, the participants in our study shared their hardships and their emotional journey to stabilising their diabetes during pregnancy, which had influenced them to rethink pursuing another pregnancy and question their ability to cope with the associated challenges [27,28]. In addition, since more than half of the participants in this study were 36 years old and above, their concern about their ability to care for young children was also closely related to their perception of being in an advanced age. Genuine apprehension is common among older mothers, even in the absence of co-morbid illnesses, because conception at an advanced age not only entails risks for the baby’s health but also adds to their worries about their own health [29]. However, all of the women of advanced age with no living child in this study still had the intention to conceive; this reflects the fact that their desire to build a family supersedes their fear of age-related complications.

This finding is not surprising because each woman, including women with diabetes, has her own reasons for wanting to conceive. Earle S from a systematic review showed that pregnancy is socially significant for some women with diabetes [18] and the desire to become a mother is an important element in the process of forming a self-identity as a woman [30]. This present study points out that motherhood not only contributes to a woman’s identity but also satisfies marital demands for continuing the family lineage from husbands and in-laws. In Asia, pregnancy is commonly bound by culture. The demand for male offspring in certain Asian cultures once women are married still carries weight even among South-East Asian women living in the West [27]. In South Asia, childbirth is described as a rite of passage for every woman [31]. This was clearly expressed by a Chinese participant, who bluntly stated that her work will be done once she gives birth to a son.

Earle S demonstrated that women with diabetes hold certain beliefs regarding fertility [18]. They consider pregnancies as fate and luck, an act connected to God and something beyond their control. In this present study, two women of the same religion referred to an ‘up to God’ belief differently in relation to their desire to have a family that fits their plans and expectations [18]. This ‘up to God’ narrative justified a participant’s decision to postpone pregnancy until her diabetes is under control, while, for another participant, it had the opposite function and served to support her pregnancy intention despite her suboptimal blood sugar control. Certain religions, such as Islam and Catholicism, encourage procreation and have produced accepted guidelines on the topic [32]. However, each encounter with a patient is unique because every woman may interpret religious teachings differently from her peers, even within the same religious circle [32]. The findings regarding the heterogeneity of practices between and within different religions and communities support Arousell’s (2016) recommendation that healthcare providers acknowledge the heterogeneity of religious beliefs in their encounters with patients, in order to ensure a successful reproductive health outcome [33]. Nevertheless, the different interpretations of the religion in reproductive health from past studies were discussed in a broader context [32,33]. From the in-depth interviews with participants in this present study, we are able to gain better understanding into the actual relations between religion, pregnancy planning and their contraceptive practice. Participants in this study put their faith in God differently; one uses faith to justify her fertility guilt while another uses it to justify her pregnancy planning. Acquiring this knowledge added more understanding why these women made decisions in their reproductive health. Hence, it is pertinent to evoke women’s religious beliefs towards procreation in order to engage them effectively in discussions on conception, PPC and contraception. In another instance, those who felt that their sexual behaviour was consistent with their religious beliefs remained silent about their pregnancy intention; hence, they were more likely to shy away from pregnancy planning and consultations with healthcare providers [34]. This may have contributed to the limited engagement with PPC that emerged from this study.

Engagement with PPC is also influenced by the relationship between women and their healthcare providers, which is crucial in ensuring successful care [35]. Participants in this study shared negative encounters with their doctors, which influenced the disclosure of their pregnancy intention. Previous studies have established that women were reticent in discussing PPC with healthcare providers because they were afraid to be judged. Providers whose attitudes were authoritative and who were not supportive of their needs, exacerbated women' fear and embarrassment [36,37]. Women reported feeling intimidated by the way healthcare providers address the issue of blood sugar levels during pregnancy [27]. Some women felt more indecisive after attending clinics that were supposed to help them plan their pregnancy; this put them at higher risk of unplanned pregnancy [38]. Women with diabetes need care plans that are personalised to their pregnancy needs and this requires healthcare providers to communicate with women effectively [28,36]. On the other hand, the healthcare system in Malaysia may not be ready to meet the demands of these women. The lack of a holistic approach in the healthcare system may be complicated by inadequate training for staff and limited facilities [28,39]. Importantly, local and international guidelines on PPC for women with diabetes do not always underline the necessity of inquiring about pregnancy intention and considering the pregnancy needs of these women [9,40].

In view of the limitations in the healthcare providers’ training on PPC and in its delivery to women with diabetes [9,28,39,40], it is not surprising that these women display a limited understanding of and engagement with PPC [37,41]. Diabetic women should receive information about healthcare during pregnancy from consultations or discussions with healthcare providers. However, this study shows that not all women with diabetes are open to approaching healthcare providers about the topic. Nevertheless, women with diabetes have reported using technology to expand their knowledge about PPC [37]. In the realm of technology, using a mobile application to support PPC delivery and maintain its effects is an acceptable and realistic approach in the West [37]. It offers avenues for ensuring continuity in the women’s engagement in their pregnancy planning and PPC. This should be further explored, as digital literacy among females in South East Asia varies, ranges from 20%–77% [42].

### Implications for practice

This study has demonstrated that women with diabetes consider several factors when deciding whether to conceive. Although many are quite similar to those that have been identified in the West, the effect of religious beliefs and cultural demands on pregnancy intention and pregnancy planning among participants in this study indicates the pressing need to improve consultations regarding PPC for women with diabetes. The clinical care of women with diabetes must be personalised and not only consider clinical, physical and biochemical stabilisation but also take into account the patients’ reproductive needs, which are subject to religious and cultural influences [34]. The provision of PPC to diabetic women must include the transmission of adequate and specific knowledge on pregnancy risks and how to reduce them, in correct and positive perspective without over-emphasising the outcomes. Studies conducted on people with diabetes and general populations conclude that planning for pregnancy is a continuum, which means that pregnancy intention may differ depending on individuals needs to procreate [43]. Therefore, regularly identifying the pregnancy needs of women with diabetes along a continuum would be pertinent. A shared agreement should be made with the women on aggressive diabetic interventions aimed at preparing them for pregnancy. Written documents on PPC care should also be improved to guide healthcare providers through administering optimum care within their limited time, facilities and busy clinics. An emphasis on partnership rather than paternalism would also enhance the quality of the relationship between healthcare providers and patients. Training the staff would be crucial to ensure that diabetic women are successfully prepared for their pregnancy.

### Strengths and limitations

Few studies have looked into pregnancy intentions among women with diabetes and, to date, even fewer have focused on Asian women. With the present study, we have contributed information on pregnancy planning among women with diabetes in more diverse ethnic groups. We demonstrated the relevance of religious and cultural beliefs as unique socio-cultural factors which make a difference in Asian women’s decisions regarding a potential pregnancy. To ensure the quality of reporting in this article, the authors have followed the COREQ check list [44]. The validity of the study was assured thanks to various measures and instruments: the conduct of all IDIs by the same researcher to ensure consistency; audio-recording; and the observation of participants’ expressions and feelings during the interviews. The use of IDIs conveyed to readers a more personal view of the women’s pregnancy intentions, which is not driven by conformity, unlike in focus group discussions. Sampling in this study was purposive and aimed to ensure maximum variation; it included women with and without pregnancy intention, and considered the participants’ various ethnicities (representing the country’s three major ethnic groups) as well as localities (urban and rural).

The internal reliability of the data was also assured. The whole team reviewed the IDIs and discussed the codes and themes over several meetings to ensure that the meanings were preserved and consistent with the study objectives. The questions, reflections and decisions on the problems that emerged during the IDIs were also discussed during the meetings, and appropriate measures were taken during the interview process. Although the semi-structured guides were based on the IM, the analysis of the data was inductive from the data itself. The results of this study should be interpreted within the context of the relevant limitations. The results only captured the experiences and perspectives of diabetic women from public primary care centres, not from hospitals or private general practitioners who may deal with more complicated diabetic cases and groups of higher socio-economic status, respectively.

## Conclusion

Our study has identified some important factors that influence the decisions that women with diabetes make regarding their pregnancy intention. We emphasised the known dilemma that women with diabetes experience when considering their desire for an ideal family structure against their perceived pregnancy risks. This study also highlighted the heterogeneity of religious beliefs, even among women of the same religion, and the impact of cultural demands on pregnancy intention. It also underlined the lack of understanding of PPC and diabetic women’s limited engagement with it. This study urges healthcare providers to employ a more personalised approach when caring for diabetic women of reproductive age. Further studies should look into strategies to improve PPC by specifically addressing the pregnancy needs, fertility-related beliefs and PPC engagement of these women.

## Supporting information

S1 Table Socio-demography and clinical profile of participants. (DOCX)

S1 Appendix The topic guide in English and Bahasa Melayu. (DOCX)

S2 Appendix The COREQ checklist of the manuscript. (DOCX)

## Abbreviations

IDI: individual in-depth interview
PCC: pre-conception care
