# Supplementary material for: Factors influencing pregnancy planning of multi-ethnic Asian women with diabetes: A qualitative study
Source: PLoS One. 2020 Dec 3;15(12):e0242690. doi: 10.1371/journal.pone.0242690 (PMC7714241; doi:10.1371/journal.pone.0242690)
Supplement: S1 Table — (DOCX) [file pone.0242690.s001.docx]

Table 1: Background data on participants.

| VARIABLE |  | N (%) |
| --- | --- | --- |
| AGE (years) | 25–30 | 4 (12.1) |
|  | 30–35 | 10 (30.3) |
|  | 36–40 | 14 (42.4) |
|  | 41–45 | 5 (15.2) |
| ETHNICITY | Malay | 19 (57.5) |
|  | Chinese | 5 (15.2) |
|  | Indian | 9 (27.3) |
| RELIGION | Islam | 19 (57.5) |
|  | Buddhism | 2 (6.1) |
|  | Christian | 3 (9.1) |
|  | Hindu | 9 (27.3) |
| PARITY | Nulliparity/No Living Child | 12 (36.4) |
|  | Multiparity | 21 (63.6) |
| LOCALITY | Urban | 17 (51.5) |
|  | Rural | 16 (48.5) |
| BLOOD SUGAR CONTROL | Yes | 9 (27.3) |
|  | NO (FBS >6.0, RBS>8.0, Hba1c >7.0) | 24 (72.7) |
| PREGNANCY INTENTION | Yes | 11 (33.3) |
|  | Ambivalence | 4 (12.1) |
|  | No | 18 (54.6) |
| FAMILY PLANNING | User | 12 (36.4) |
|  | Non-User | 21 (63.6) |
