# Supplementary material for: Factors influencing pregnancy planning of multi-ethnic Asian women with diabetes: A qualitative study
Source: PLoS One. 2020 Dec 3;15(12):e0242690. doi: 10.1371/journal.pone.0242690 (PMC7714241; doi:10.1371/journal.pone.0242690)
Supplement: S1 Appendix — (DOCX) [file pone.0242690.s002.docx]

**S1_Topic guide_english and Bahasa Malaysia**

**UNDERSTANDING PREGNANCY PLANNING OF WOMEN WITH DIABETES.**

**Introduction:**

- Ice breaking
- Greetings
- Reinforce confideniality

| English | Bahasa Malaysia |
| --- | --- |
| 1. Now that you are still capable of getting pregnancy, do you have any intention for pregnancy? Why? 2. What influence your decision to conceive or avoid it? 3. If nulliparous , go to Q4.   When did you last conceive?  Can you tell us your experience during the last pregnancy with diabetes?  Prompt:   - - feeling   - challenges.   - How did you plan the last pregnancy? (past PPC)  1. Regarding your pregnancy planning; is there anything that influence your decision or strategies to conceiving?  - If no pregnancy intention; what do you do to avoid pregnancy? Prompt: contraception - If has pregnancy intention; What do you do to conceive? (current PPC)   Prompt: does having diabetes affect your planning?   1. What do you know about pregnancy with diabetes?   Prompt: the effects?  If no risk is mentioned; prompt: Do you know any risk if you are pregnant with diabetes?   1. What do you understand about pre pregnancy care? | 1. Buat masa ni, puan masih boleh hamil. Ada niat untuk hamil? Kenapa?  2. Apa yang mempengaruhi keputusan untuk hamil atau pun mengelakkan kehamilan?  3. Kalau nulliparous , terus ke Q4.  Bila kali terakhir hamil?  Bagaimana pengalaman mengandung dengan diabetes?  Prompt:   - perasaan - Halangan. - Bagaimana puan merancang kehamilan yang lepas?   4. Apakah yang mempengaruhi rancangan atau strategy puan untuk hamil atau tidak?   - Kalau tiada niat hamil, bagaimana puan hendak mengelakkan hamil? Prompt: contraception - Kalau ada niat hamil, apa strategy puan untuk hamil? (current PPC)   Prompt: adakah mempunyai diabetes mempengaruhi rancangan puan?  5. Apa yang puan tahu diabetes?  Prompt: kesan diabetes?  Kalau tdak tahu; prompt: Ada risiko ke bila hamil dengan diabetes?  6. Apa pendapat puan tentang penjagaan sebelum kehamilan? |
