# Supplementary material for: Factors influencing pregnancy planning of multi-ethnic Asian women with diabetes: A qualitative study
Source: PLoS One. 2020 Dec 3;15(12):e0242690. doi: 10.1371/journal.pone.0242690 (PMC7714241; doi:10.1371/journal.pone.0242690)
Supplement: S2 Appendix — (DOCX) [file pone.0242690.s003.docx]

Appendix S2: COREQ checklist

| **Domain 1: Research team and reflexivity** | |
| --- | --- |
| 1. Interviewer/facilitator | One interviewer carried out the interviews: |
| 1. Credentials | NCJ, NH, LPY ae professional researchers (NCJ and LPY are professors of Family Medicine, NH are PhD holders, II is currently pursuing PHD. |
| 1. Occupation | All interviewers are lecturers with University Malaya or University Putra Malaysia |
| 1. Gender | NCJ is male and the others are female. |
| 1. Experience and training | NCJ, LPY, NH are experienced qualitative interviewers. II has undergone basic and advance qualitative training |
| 1. Relationship established | No prior relationship between the interviewers and interviewees existed. |
| 1. Participant knowledge of the interviewer | Potential interviewees were informed about the study prior to the interview, with the study information sheet approved by the research ethics committee (NMRR ID: 16-385-29240). |
| 1. Interviewer characteristics | All interviewers, and members of the wider research team, had pre-existing interests in diabetes. II, NH had a long-standing interest in pre-pregnancy care. |
| **Domain2: Study design** | |
| 1. Methodological orientation and theory | Qualitative methods are used to gain firsthand knowledge of participants’ experience and perceptions of pregnancy planning. The interviews were guided by a semi-structured protocol drawn based on a combination of the theory of reasoned action and theory of planned behaviour |
| 1. Sampling | Purposive sampling: women with diabetes from age 18-45 of different ethnicity were identified and contacted. |
| 1. Method of approach | Initial contact was by personal face to face approach |
| 1. Sample size | 40 women were approached personally but 33 in depth interviews were achieved after saturation achieved. |
| 1. Non-participation | Five were excluded because of menopause |
| 1. Setting of data collection | Diabetic clinic of 2 rural and 2 urban primary care clinics |
| 1. Presence of non-participants | Two participants refused to participate |
| 1. Description of sample | Information about age and women’s background were displayed in S1 |
| 1. Interview guide | Topics of topic guide are listed in the main text. |
| 1. Repeat interviews | No repeat interview done. |
| 1. Audio/visual recording | Interviews were audio-recorded. |
| 1. Field notes | Brief field notes were kept by interviewer |
| 1. Duration | Interviews lasted between 40 and 60 minutes |
| 1. Data saturation | The data was saturated at participant number 31 and stopped recruiting at participant number 33. |
| 1. Transcripts returned | 12 transcripts were not returned to interviewees. |
| **Domain 3: Analysis and findings** | |
| 1. Number of data coders | II was the primary coder, Coding consistency was co-checked by the all interviewers. |
| 1. Description of coding tree | Codes represented distinct viewpoints on each topic. |
| 1. Derivation of themes | Themes were closed related to the subject matter of the topic guide. |
| 1. Software | NVivo 10 |
| 1. Participant checking | Members’ check were done |
| 1. Quotations presented | Yes |
| 1. Data and findings consistent | We have attempted to present our findings in a clear manner, consistent with the data collected |
| 1. Clarity of major or minor themes |  |
